# Artesunate Dry Emulsion Formulation Combined with Antibiotics for Treatment of *Helicobacter pylori* Infections: In Vitro/In Vivo Evaluation

**DOI:** 10.3390/ijms241311008

**Published:** 2023-07-02

**Authors:** Canh Le-Tien, Lindsay Blemur, Dennis Baltzis

**Affiliations:** Solstar Pharma, Department of Chemistry, 237-43 Boulevard Samson, Laval, QC H7X 3R8, Canada; lindsay.blemur@solstarpharma.com

**Keywords:** *Helicobacter pylori*, gastroduodenal ulcers, stomach cancer, antibiotic resistance, artesunate, artesunate dry emulsion formulation, antibacterial, combination therapy, in vitro/in vivo evaluation

## Abstract

*Helicobacter pylori* is the primary pathogen responsible for causing gastroduodenal ulcers and stomach cancer. The standard treatment for *H. pylori* typically involves a combination of antibiotics and acid-reducing medications. However, the recurrence of ulcers is closely linked to the emergence of antibiotic resistance in *H. pylori*, necessitating the development of alternative drugs. This report focuses on the investigation of artesunate as a potential alternative to reduce antibiotic use and enhance effectiveness against *H. pylori*. Unfortunately, commercial artesunate is available in an acid form, which has poor solubility, especially in gastric acid fluid. The aim of this study is to utilize a water-soluble formulation of artesunate called dry emulsion formulation (ADEF) and combine it with amoxicillin to eradicate *H. pylori*. In vitro studies were conducted to evaluate the activity of ADEF against *H. pylori* and determine its inhibitory concentrations. In addition, pharmacokinetic parameters of orally administered ADEF and native artesunate were investigated in rats for in vivo studies. The results showed that when combined with amoxicillin and pantoprazole, ADEF exhibited effectiveness against *H. pylori*. It is worth noting that the solubility of ADEF in gastric acid appears to be a critical factor for achieving successful treatment. Consequently, ADEF could be considered a promising candidate for *H. pylori* therapy.

## 1. Introduction

*Helicobacter pylori* is a gram-negative bacterium that infects the epithelial lining of the stomach and persists in the absence of treatment. It is estimated that there are approximately 4.4 billion individuals with *H pylori* infection worldwide [1]. Although most infected patients are asymptomatic, *H. pylori* is a major cause of peptic ulcer disease and chronic gastritis. Persistent infection with *H. pylori* increases the risk of developing gastric cancer and gastric mucosa-associated lymphoid tissue lymphoma in adults [2]. *H. pylori* may also have a role in uninvestigated and functional dyspepsia, unexplained iron deficiency anemia [3], and idiopathic thrombocytopenic purpura [4]. Many gastrointestinal diseases infected with *H. pylori* will develop clinically significant sequelae, such as peptic ulcer disease and gastric cancer [2].

First-line treatments to eradicate *H. pylori* are based on triple or quadruple therapy, including generally proton pump inhibitor (PPI), amoxicillin, clarithromycin, or metronidazole and bismuth salt. However, their effectiveness is diminishing due to the rise in antibiotic resistance, specifically the increasing resistance of *H. pylori* to amoxicillin and tetracycline [5]. Some of these strains are resistant to clarithromycin [6], metronidazole, and levofloxacin [7,8]. Recently, a new commercial formulation called Talicia has been introduced, which combines rifabutin with amoxicillin and omeprazole (PPI) to eradicate *H. pylori*. While rifabutin has proven effective, there are concerns about whether its widespread use will contribute to the development of resistance to rifamycin among tuberculosis strains [9].

Since antibiotic resistance constitutes a serious threat to human health, there is an urgent need to find solutions to limit the emergence of antibiotic resistance. For the moment, the use of alternative non-antibiotic bioactive compounds may successfully achieve the therapeutic goal of eradication without undue risks. Several natural health products, such as essential oils [10] or artemisinin and its derivatives [11], were proposed as an alternative to treat *H. pylori* infection. It is of interest to mention that artemisinin and its derivatives can eradicate *H. pylori* and prevent gastric cancer [12]. Despite the promising results of these products in vitro, no in vivo study has been conducted thus far to confirm their effectiveness.

**Figure 1:**
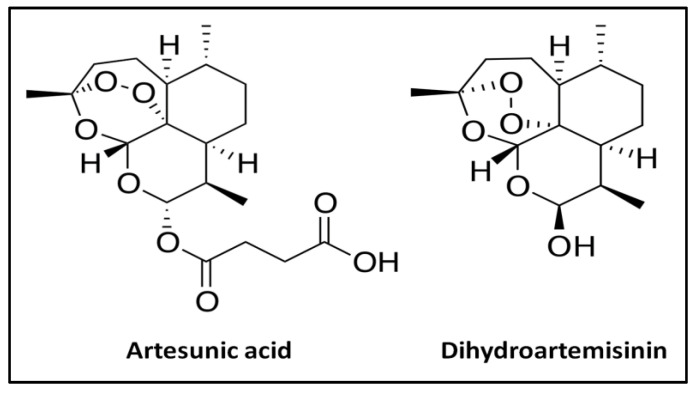
Chemical structure of artesunate and its metabolite, dihydroartemisinin.

Artemisinin is a sesquiterpene lactone endoperoxide [13] extracted from the plant *Artemisia annua,* which has been used in China for the treatment of malaria for over a thousand years [14]. To improve its bioavailability, several derivatives from artemisinin, such as artemether, arteether, and artesunate, were approved by the World Health Organization (WHO) and are currently used to treat malaria. After oral administration, these derivatives are hydrolyzed within minutes to their mainly active metabolite dihydroartemisinin (DHA, Figure 1), which is responsible for the biological activity. Artesunate is a hemi-succinate derived from artemisinin. Commercial artesunate is available in the form of a dry artesunic acid powder (pH 3.0–5.0) which is poorly soluble in aqueous medium, particularly in gastric acid fluid [15]. According to the Drug Bank [16], the water solubility of artesunate in acidic form is about 0.68% (*w*/*w*). Additionally, artesunate is unstable in aqueous, acidic, and basic media [17]. After oral administration, artesunate generally remains insoluble in gastric acid and is rapidly hydrolyzed into dihydroartemisinin (DHA). It is worth mentioning that DHA is less stable and readily decomposes into the inactive product 2-deoxyartemisinin [18,19,20].

In this context, it is important to develop a stable and soluble form of artesunate that can effectively exert its antibacterial activity in the acidic environment of the stomach. This feature is crucial for achieving a successful treatment outcome. The primary objective of this report is to explore the potential use of artesunate as an alternative to reduce the reliance on antibiotics in triple- or quadruple-therapy regimens, considering the emergence of resistant strains. Specifically, a soluble form of artesunate, prepared using dry emulsion formulation (ADEF), was combined with amoxicillin (Amox) and pantoprazole (a proton pump inhibitor, PPI) to combat *H. pylori* infection. The ADEF formulation was thoroughly characterized, and the inhibitory concentrations (IC50 and IC90) of ADEF against different *H. pylori* strains were determined. Additionally, the pharmacokinetic parameters of ADEF were investigated in rats for in vivo studies, and its efficacy, both alone and in combination with Amoxicillin and PPI, was evaluated in mice. The novelty of this work lies in the use of dry emulsion technology to create a water-soluble form of artesunate. This allows the drug to dissolve effectively in gastric acid fluid, enhancing its antibacterial activity in the stomach. By using artesunate as an alternative, it may help reduce antibiotic resistance and minimize side effects. However, to date, no in vivo study has been conducted to confirm the efficacy of water-soluble artesunate combination therapy.

## 2. Results and Discussion

### 2.1. Characterization of Artesunate Dry Emulsion Formulation

#### 2.1.1. Solubility

As previously mentioned, native artesunate did not dissolve in simulated gastric fluid (SGF) with a pH of 1.5 even after 30 min of stirring. Instead, a solid precipitate quickly formed when the agitation ceased. In contrast, ADEF powders exhibited solubility in SGF, and the resulting solution remained stable for at least 30 min under continuous stirring.

#### 2.1.2. FTIR Analysis

**Figure 2:**
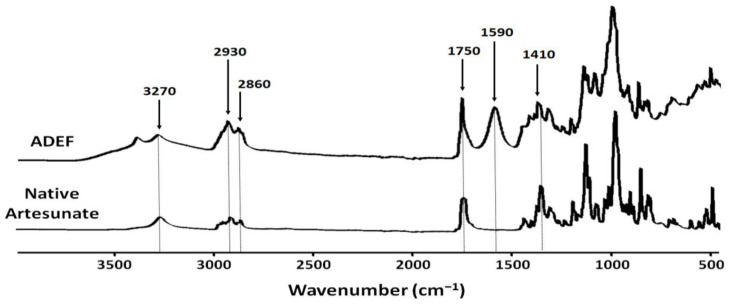
FTIR spectra in the form of native artesunate and artesunate dry emulsion formulation (ADEF) powders.

For native artesunate (Figure 2), absorption bands located in the spectral region of 2930–2860 cm^−1^ are assigned to alkyl (C-H) stretching vibrations of the alkyl chain from sesquiterpene structure. Typically, absorption bands for artesunate are located at 1750 and 1420 cm^−1^, assigned to carboxylic group (protonate form, -COOH) from succinic acid residues.

According to the provider, the water-soluble artesunate formulation is a complex of carboxymethylcellulose and emulsifying agents. Referring to ADEF spectrum, artesunate was mainly stabilized with the carboxymethyl cellulose (CMC) matrix. For this reason, there is an increased intensity of the absorption bands located in the spectral region of 2930–2860 cm^−1^ assigned to alkyl (C-H) stretching vibrations of the alkyl chain mainly from sesquiterpene of artesunate and the emulsifying agent. An absorption band at 1750 cm^−1^ was attributed to carboxylic acid (protonated form, -COOH) group mainly from artesunic acid. Additionally, absorption bands 1590 and 1410 cm^−1^ were assigned to carboxylate (deprotonated form, -COO ^−^) group from CMC and artesunate. These results clearly indicate that ADEF possesses two carboxyl forms. One is under in the form of carboxylate salt (-COO ^−^) from CMC, and the other is in the form of carboxylic acid (-COOH) form basically from artesunate. These analyses suggest that the solubility of ADEF could be due in part to the carboxylate group of CMC stabilizing with artesunate.

#### 2.1.3. X-ray Diffraction

**Figure 3:**
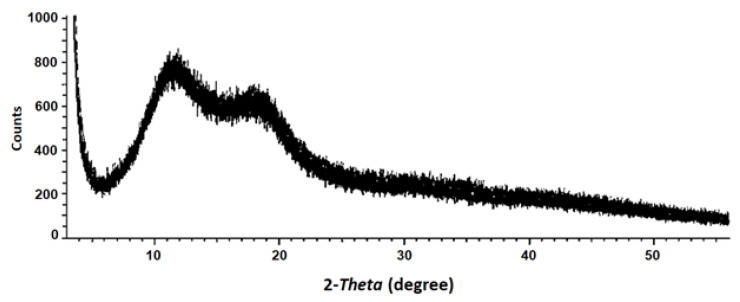
X-ray diffraction of artesunate dry emulsion formulation (ADEF).

The X-ray diffractogram of ADEF powders (Figure 3) showed that bands located at the 2-Theta angle corresponding to 12° and 18° were broader and had moderately lower intensities, suggesting a lesser crystalline structure (or amorphous structure). This phenomenon could be due to the presence of artesunic acid, which disorganizes the crystalline structure of CMC.

#### 2.1.4. SEM Analysis

**Figure 4:**
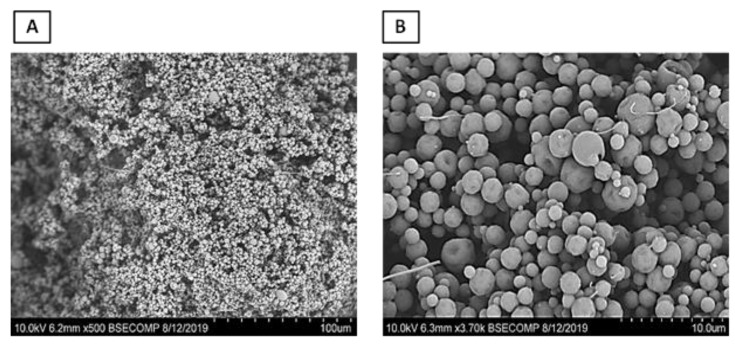
Scanning electron microscopy of artesunate dry emulsion formulation at magnifications of 500× (**A**) and 4000× (**B**).

The scanning electron microscopy (SEM) micrographs (Figure 4) revealed that the ADEF granules exhibited a generally spherical shape with moderately small, finely textured surfaces. The size of the granules varied between 10 and 80 μm.

### 2.2. In Vitro Study

#### 2.2.1. Determination Antibacterial Activity of Native Artesunate and ADEF via Agar Disk Diffusion Assay

**Figure 5:**
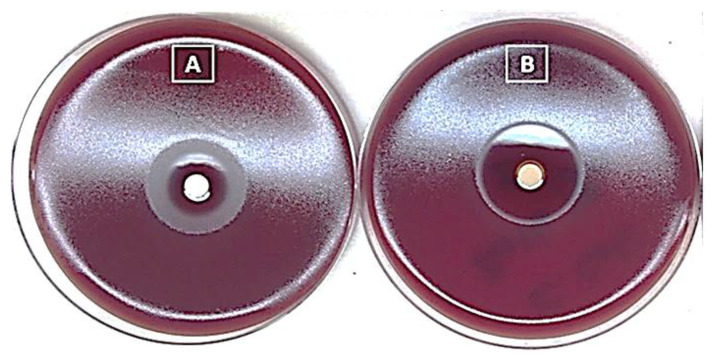
Agar disk diffusion assay for native artesunate (**A**) and artesunate dry emulsion formulation (**B**) against *Helicobacter pylori*.

In general, the results obtained (Figure 5) demonstrate the effectiveness of both native artesunate and ADEF against *H. pylori*. However, the inhibition zone observed for ADEF was clearer and larger compared to that of native artesunate. It is important to note that two distinct zones were observed for native artesunate, which can be attributed to its low solubility. The clear interior zone corresponded to the effective zone, resulting from the solubility of native artesunate. In contrast, the exterior zone appeared foggy, likely caused by the insoluble portion of native artesunate.

Based on these findings, ADEF was chosen for further advanced studies. Previous research suggests that the antibacterial activity of artesunate is likely attributed to its ability to generate reactive oxygen species through the cleavage of the endoperoxide bridge in the 1,2,4-trioxane scaffold [21].

#### 2.2.2. Determination of Inhibitory Concentration

The broth dilution method was employed to determine the MIC values, given its widespread usage for this purpose. The method involved creating a series of dilutions of ADEF in a liquid growth medium and introducing a standardized inoculum of *H. pylori* (10^5^ cfu/mL). The MIC value was identified as the lowest concentration of ADEF that completely inhibited bacterial growth, which was determined to be an average of 5 μg/mL. While there is no available literature specifically addressing artesunate’s efficacy against *H. pylori*, previous investigations on artemisinin and its derivatives have indicated inhibitory concentrations ranging from 0.5 to 10 μg/mL [22], aligning with our findings.

**Figure 6:**
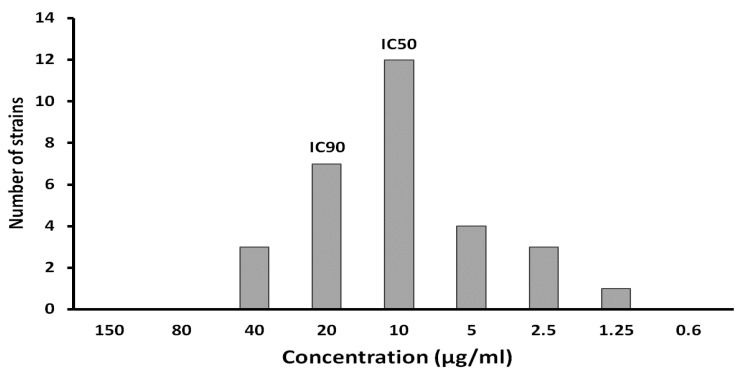
IC50 and IC90 of artesunate dry emulsion formulation were determined from 30 strains of *Helicobacter pylori* with different origins.

The potential of ADEF to inhibit *H. pylori* was evaluated using a plate dilution assay involving 30 strains of *H. pylori* from diverse sources. The results demonstrated that ADEF effectively suppressed the growth of multiple *H. pylori* strains, displaying high antimicrobial activity in vitro (Figure 6). The 50% and 90% inhibitory concentrations of ADEF against *H. pylori* were determined to be 10 and 20 μg/mL, respectively.

### 2.3. In Vivo Study

#### 2.3.1. Pharmacokinetic of ADEF

Understanding the pharmacokinetic (PK) parameters of ADEF is crucial for investigating the development and efficacy of soluble artesunate in the gastric medium against *H. pylori*. Following oral administration, artesunate is rapidly absorbed, but it undergoes significant hepatic first-pass metabolism, leading to the formation of its principal active metabolite, DHA. This metabolic process is likely mediated by CYP2A6, which serves as the primary metabolizing enzyme [23].

The results indicated that artesunate was rapidly absorbed and exhibited a dose-dependent increase. Following administration of ADEF (Table 1), artesunate levels reached their maximum concentration (Tmax) within 5 min, whereas native artesunate showed a Tmax of 15 min. Notably, the maximum concentration (Cmax) of ADEF reached a higher value of approximately 1890 ng/mL compared to native artesunate, which reached 701 ng/mL at a dosage of 150 mg/kg. It is worth mentioning that there was an approximately 2.7-fold improvement in the bioavailability of artesunate in the context of ADEF administration.

In terms of DHA (Table 2), it was observed in the plasma with a Tmax of 10 min for lower doses (25–50 mg/kg of ADEF) and 15 min for higher doses (100 and 150 mg/kg). In contrast, for native artesunate, DHA was detected with a longer Tmax of 45 min. Comparing the Cmax values after a dose of 150 mg/kg, DHA derived from ADEF reached 3870 ng/mL, which was higher than the 1450 ng/mL observed with native artesunate. Additionally, ADEF exhibited a 2.7-fold improvement in bioavailability. Importantly, throughout this experiment, no mortality or abnormal signs were observed following the single oral gavage administration of different doses of native artesunate or ADEF.

**Figure 7:**
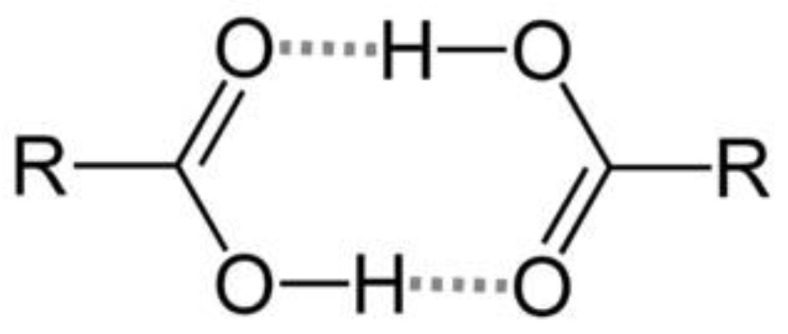
Stabilization by dimerization of carboxylic acid groups via hydrogen-bonding.

Native artesunate is commonly available in the form of acid powder in the commercial market, primarily due to its storage stability. This stability is believed to stem from its capacity to undergo dimerization through inter-molecular hydrogen bonding between carboxyl groups (as shown in Figure 7). Consequently, artesunate exhibits limited solubility, particularly in gastric acid, owing to the protonation of the majority of its carboxyl groups.

Unlike native artesunate, ADEF was developed using emulsions, resulting in its solubility being unaffected by pH. This characteristic accounts for its rapid absorption and enhanced bioavailability. Consequently, ADEF demonstrates superior antibacterial activity compared to native artesunate as confirmed by the agar disk diffusion assay.

#### 2.3.2. In Vivo Efficacy of ADEF against *H. pylori*

**Figure 8:**
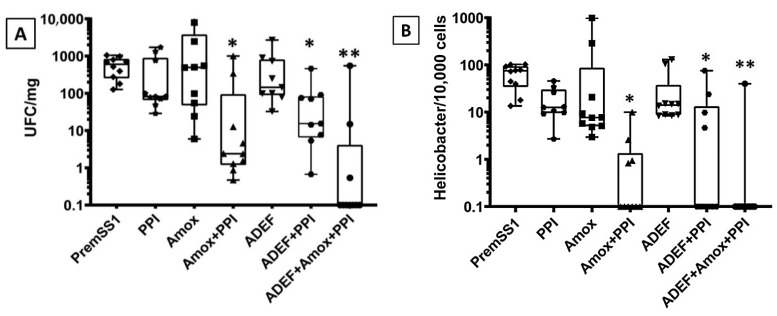
Boxplot graphical representation of results obtained from bacterial culture (**A**) and qPCR (**B**) of *H. pylori* extracted from the mouse stomach. Box boundaries indicate the first and third quartiles. The median (50%) is given as the horizontal line within the box. * *p* < 0.01 and ** *p* < 0.001 (Mann–Whitney test) are significant difference between untreated mice and different experimental conditions.

The effectiveness of ADEF against *H. pylori* has been investigated in C57BL/6 mice infected with PremSS1. The recommended treatment involves administering a single dose of ADEF (40 mg/kg of artesunate) along with amox (30 mg/kg) and PPI (150 mg/kg) for a duration of 14 days. The results indicated that administering PPI, amox, or ADEF individually did not exhibit a significant effect against *H. pylori* in infected mice (Figure 8A,B). It is evident that PPI, being an antacid, lacks antibacterial properties. Conversely, although amox is an antibiotic, it demonstrates limited efficacy when used in isolation without PPI. However, when PPI and amox are combined, a noteworthy inhibition of *H. pylori* growth is observed. Therefore, PPI may play a crucial role in eradicating *H. pylori* by reducing gastric acid production, which potentially enhances antibiotic stability or inhibits *H. pylori* urease, as suggested by a particular author [24]. Similarly, ADEF alone did not exhibit any effect, but when combined with PPI, it displayed a significant inhibition of bacterial growth.

In terms of microbial quantification (Figure 8A), the sensitivity is typically lower compared to PCR quantification, likely due to potential contamination from other bacteria during cell and medium manipulation. Currently, the utilization of PPI in combination with amox and ADEF has demonstrated an average efficacy eradication rate exceeding 70%. For PCR quantification analysis (Figure 8B), this method offers greater accuracy compared to microbial quantification, as it is more sensitive and specific in detecting *H. pylori*. In fact, an eradication rate of approximately 90% for *H. pylori* was observed.

Artesunate, initially commercialized as artesunic acid with poor solubility, underwent reformulation using dry emulsion technology to enhance its solubility and stability, especially in gastric acid fluid. The reformulated artesunate, known as ADEF, exhibited superior bioavailability and efficacy compared to the native artesunate, as indicated by in vitro results.

Interestingly, in vitro studies demonstrated the high effectiveness of artesunate against *H. pylori*. This could be attributed primarily to the favorable test conditions typically provided in laboratory settings, which are optimized for bacterial growth. Moreover, the medium employed, commonly a «poly-house-made» medium, maintains a neutral pH range of 7.0 to 7.4, which optimizes the solubility of artesunate.

In contrast, in vivo experiments are carried out within living organisms, such as mice in this scenario, involving intricate interactions between the drug (including its pharmacokinetics, metabolism, and bioavailability), the host (including the host immune response), and the pathogen (including virulence factors). Moreover, *H. pylori* typically colonizes the acidic environment of the human stomach, where pH values range from 1.5 to 2.0. Within this environment, gastric acidity significantly impacts the solubility of artesunate and may lead to its degradation or inactivation. Consequently, the inclusion of PPI in the treatment combination becomes necessary to reduce stomach acid levels, thereby preventing the degradation of artesunate and enhancing its effectiveness.

Considering both in vitro and in vivo data is crucial for assessing the activity of artesunate against *H. pylori*. Each type of study provides distinct insights into the drug’s mechanism of action and potential clinical usefulness.

Artesunate, derived from artemisinin found in *Artemisia annua*, has been utilized in China for over 2000 years to treat malaria. Recently, the Food and Drug Administration (FDA) approved artesunate for the treatment of severe malaria in both adult and pediatric patients [25]. Previous studies [21,26] have indicated that artemisinin and its derivatives exhibit in vitro antibacterial properties against *H. pylori*. However, there have been no confirmed in vivo studies conducted thus far. In vivo studies can be challenging, expensive, and complex, involving tasks, such as culturing *H. pylori*, infecting and caring for animals, and requiring specialized expertise. Nevertheless, it has been suggested that the endoperoxide bridge present in artesunate acts as a pro-oxidant, generating reactive oxygen species (ROS) that contribute to its antibacterial activity.

At present, the current approach to triple or quadruple therapy involves combining PPI and amoxicillin with other antibiotics, like clarithromycin, erythromycin, metronidazole, and—more recently—rifabutin (Talicia). Nonetheless, employing antibiotics comes with numerous drawbacks:

- Side effects caused by excessive and high dose of antibiotics
  The present treatment regimen for *H. pylori* infection involves a high dosage (amoxicillin 1000 mg and clarithromycin 500 mg) administered two or three times a day over an extended period, typically 14 days [27,28]. Common antibiotic side effects encompass rash, dizziness, nausea, and diarrhea among others. Notably, more severe reactions may include the development of yeast infection, resulting in diarrhea that can potentially lead to severe colon damage and even death [29]. Additionally, in rare instances, myelotoxicity represents the most notable adverse effect associated with rifabutin [30];
- Emergence of antibiotic resistance
  Excessive use of antibiotics can result in the emergence of antibiotic resistance, making the treatment of bacterial infections more challenging. A meta-analysis conducted in 2018 [31] revealed that *H. pylori* has surpassed the recommended threshold for resistance to clarithromycin, levofloxacin, and metronidazole, as outlined in the Maastricht-V consensus. In numerous regions across the globe, there is a prevalent high resistance rate (15%), while certain areas exhibit dual resistance rates exceeding 15% for clarithromycin and metronidazole. Conversely, resistance rates for amoxicillin and tetracycline remain low. As for rifabutin, its current resistance rate is minimal. However, the extensive utilization of rifabutin for eradicating *H. pylori* in individuals infected with mycobacterium tuberculosis could contribute to the development of antibiotic resistance [32];
- High cost
  Triple or quadruple therapy can pose a significant financial burden, particularly in regions without accessible or subsidized healthcare. Consequently, this expense becomes a formidable barrier for numerous patients unable to bear the treatment’s cost;
- Oral antibiotics induced changes in the human gastrointestinal microbiota
  The administration of antibiotics frequently leads to significant changes in the gut microbiota [33,34], necessitating the use of probiotics to restore the microbial community.

Due to the benefits mentioned above, the utilization of artesunate is intriguing as it can decrease susceptibility to antibiotics and serve as a substitute for those that have developed resistance. The utilization of an artesunate-based eradication regimen proves to be a meaningful and secure approach for eliminating *H. pylori*. At present, the suggested formulation, when combined with ADEF, is orally administered in a single dose per day for 14 days, effectively eradicating these bacteria. It is important to note that artesunate not only demonstrates efficacy against *H. pylori* but also exhibits the ability to prevent *H. pylori*-induced gastric carcinogenesis [12,35]. Furthermore, artesunate does not impact the gut microbiota [36], which provides an advantage over antibiotics in inhibiting *H. pylori* infection.

## 3. Materials and Methods

### 3.1. Reagent

Native (unmodified) artesunate was purchased from Huvepharma Italia s.r.l. (99%, Garessio, Italy), and artesunate dry emulsion powder (60%, *w*/*w*) was provided by Solstar Pharma (Montreal, QC, Canada). *H. pylori* PremSS1 strain (supplied by Dr Anne Mueller, University of Zürich, Zürich CH, Switzerland) was used. The functionality of the *cag* pathogenicity Island (*cag*PAI) was verified in vitro in a coculture model with the AGS gastric epithelial cells induction of a so-called «hummingbird» phenotype. Muller Hilton medium from bioMérieux (Craponne, France), amoxicillin from Laboratoire PanPharma (Beignon, France) and pantoprazole from Laboratoire Arrow Générique (Bordeaux, France). The other chemicals were reagent-grade and used without purification.

### 3.2. Characterization of Artesunate Dry Emulsion Formulation (ADEF)

#### 3.2.1. Solubility

An amount of 0.5 g of native artesunate and ADEF was dispersed in 10 mL of simulated gastric fluid (SGF, pH 1.5). All solutions were incubated at 36.5 °C under mild shaking (100 rev/min) in a G24 Environmental Incubator Shaker (New Brunswick Scientific Co., Edison, NJ, USA) for 30 min.

#### 3.2.2. Fourier-Transform Infrared (FTIR) Analysis

FTIR spectra were recorded with a Spectrum OneTM (Perkin-Elmer Instruments, Norwalk, CA, USA) equipped with a universal attenuated total reflectance (UATR) device for powder analysis on the spectral region 4000–650 cm^−1^ with 24 scans at 4 cm^−1^ resolution. Native artesunate and ADEF were directly analyzed in powder form, and all obtained spectra were normalized over the range using the SpectrumTM software 3.02.

#### 3.2.3. X-ray Diffraction

The polymorphism of ADEF was evaluated by X-ray diffractometry (X-RD, Karlsruhe, Germany) with Cu Kα radiation operating at wavelength of 1.5406 Å. The XRD patterns were recorded between 5 and 50° (2-theta angle).

#### 3.2.4. Scanning Electron Microscopy (SEM) Analysis

The morphology and surface characteristics of ADEF were examined at magnifications of 500× and 4000× with a S-3400N Variable Pressure SEM (JEOL Ltd., Tokyo, Japan). The images were obtained with voltages of 10 kV and high vacuum.

### 3.3. In Vitro Study

#### 3.3.1. Agar Disk Diffusion Assay

Practically, impregnated paper disks with a determined concentration (~20 mg/mL) of ADEF were deposited on the surface of a standardized culture medium previously inoculated with a calibrated inoculum of a pure culture of *H. pylori*. After incubation, the Petri dishes were examined, and the diameters of the zones of inhibition surrounding the disks were measured. The obtained values were thereafter compared to the critical values of the different antimicrobial standards to determine the clinical categorization (resistant, intermediate, sensitive).

#### 3.3.2. Determination of Inhibitory Concentrations

In total, about 30 strains of *H. pylori* were tested. These *H. pylori* strains were isolated from gastric biopsies from different geographic origins: Africa (2 from Algeria and 5 from Congo), Asia (4 from Japan), America (2 from Costa Rica), and 17 strains isolated in France. The identities of *H. pylori* strains to test were 3392, 3995, 3997, 4001, 4010, 4011, 4013, 4015, 4030, 4032, 4037, 4038, 4039, 4063, 4064, LB016, ALG126, ALG140, AFR65, AFR69, AFR73, AFR76, AFR93, CR990922NN, CR9909276, JAP09236, JAP09244, JAP09260, JAP TH79 and CCUG17874. All these strains were provided by the Centre National de Reference (C.N.R) and the Centre Hospitalier Universitaire (CHU) de Bordeaux (Amélie Raba-Léon place, Bordeaux Cedex).

The antibacterial activity of ADEF was performed by measuring values of inhibitory concentrations of various *H. pylori* strains. In the present study, it is of interest to determine the IC50 and IC90, which represent the concentrations of ADEF required to inhibit 50% and 90% of *H. pylori* growth, respectively. These inhibitory concentrations were carried out by using the agar dilution method with the following parameters:

- Bacterium inoculum: a suspension of approximately 10^5^ cfu/mL is prepared in brucella broth from a 48 h culture grown at 37 °C in microaerobic conditions;
- Culture medium: Mueller–Hinton containing sheep blood with globular extract (10%);
- Sample preparation: Starting with a stock solution of ADEF at a concentration of 2 mg/mL, suitable dilutions were made using sterile distilled water (200 µL). These dilutions were then added to a plate containing 39.8 mL of culture medium to create a range of final concentrations from 0.6 to 1250 µg/mL. For each of the 30 *H. pylori* strains, duplicate samples were prepared;
- Inoculation: the inoculation was performed with a multiple inoculator, and the plates were incubated for 48 h at 37 °C in a workstation containing a microaerobic atmosphere (5% O_2_, 10% CO_2_, 85% N_2_). Double replicate assays were carried out to ensure the reproducibility of results. The minimum inhibitory concentration (MIC) is determined as the lowest concentration of the bioactive agent which inhibits the growth of the *H. pylori* strain.

### 3.4. In Vivo Study

All animals utilized in this study were treated in compliance with the guidelines provided in the latest edition of the Guide to the Care and Use of Experimental Animals published by the Canadian Council on Animal Care as well as the Guide for the Care and Use of Laboratory Animals. The Animal Care Committee (ACC) of ITR Laboratories Canada thoroughly reviewed and evaluated the protocol and any applicable revisions. The ACC’s approval of the study plan and amendments is documented and maintained on file at ITR (Urfe Bay, QC, Canada).

#### 3.4.1. Pharmacokinetic Parameter Determination

The objective of this study was to investigate the pharmacokinetic (PK) profile of ADEF after a single oral (gavage) administration to rats at the following doses:

- Low dose: 25 and 50 mg/kg of ADEF;
- Mid dose: 100 mg/kg of ADEF;
- High dose: 150 mg/kg of ADEF.

For comparative study, native artesunate was also included in this study, but only at a high dose of 150 mg/kg.

Indeed, 36 male Sprague Dawley rats (7–8 weeks old, average weight approximately 250 g) were housed in groups of up to three in bins (at 21 ± 2 °C) equipped with an automatic watering system and feed ad libitum (Envigo Global 18% protein rodent diet #2018C). After a 1-week acclimation period, ADEF was administered orally to rats, and blood samples were collected at various time intervals (5, 10, 15, 30, 45, and 60 min) using K2EDTA tubes as anticoagulants. The collected tubes were then placed on wet ice. Subsequently, the samples were centrifuged at 1000× *g* for 10 min at 4 °C. The resulting plasma was recovered and stored frozen at temperatures below or equal to −60 °C for further analysis. The determination of artesunate and its metabolites (DHA) in the plasma was performed using liquid chromatography coupled with a triple quadrupole Shimadzu™ LC-MS 8030. For additional details, please refer to the Supplementary Materials section, specifically Tables S1–S3.

Non-compartmental analysis of the test item and its metabolite concentrations in plasma was performed by using the Phoenix^®^ WinNonlin^®6^ 6.4 software.

#### 3.4.2. In Vivo Efficacy of ADEF against *H. pylori*

The experiments were conducted at the University of Bordeaux in dedicated pathogen-free animal facilities. These animal experiments adhered to the European Union recommendations (European Directive 2010/63/EU) regarding animal experimentation. The project underwent evaluation by the local ethical committee of the University of Bordeaux and complied with the French Ministry of Agriculture Guidelines on Animal Care and the French Committee of Genetic Engineering. Throughout the study, the principle of the three Rs (replacement, reduction, and refinement) was upheld.

Animals: A mouse model of *H.-pylori*-induced disease using pre-murine Sydney strain-1 (PremSS1) in mice C57BL/6 was extensively used in *Helicobacter* research. The persistence of mouse colonization, distribution, and long-lasting infection is attributable to the strain’s capabilities. For these reasons mice C57BL/6 were selected in this study. In fact, male mice with «specific pathogen-free» status (CHU of Bordeaux, Amélie Raba-Léon place, Bordeaux Cedex) at 6 weeks of age were housed in an acclimatization room for 1 week before experimentation.

*H. pylori* strain: The *H. pylori* PremSS1 strain was used, and the functionality of the *cag* pathogenicity island was verified in vitro in a coculture model with the gastric epithelial line AGS “Hummingbird” phenotype.

Mouse gavage with *H. pylori*: Mice were fasted the day before the gavage days. They were force-fed with approximately 10^8^ *H. pylori* PremSS1 in 100 µL of phosphate-buffered saline in the morning and then placed in a cage for the rest of the day under normal conditions. The gavages were carried out on three consecutive days. A bacteria viability test was accomplished post-gavage by platting a bacterial pellet on «Poly-house-made» agar for 24 h. This culture medium was prepared in the laboratory (Wilkins Chalgren medium enriched with 10% human blood and made selective by the addition of vancomycin 10 µg/mL, trimethoprim 5 µg/mL, amphotericin-B 1 µg/mL and cefsulodin 2 μg/mL).

Culture and identification: After pouring in «Poly-house-made», *H. pylori* PremSS1 strain was collected after 24 h incubation in a microaerobic atmosphere at 37 °C. *H. pylori* were identified by its phenotypic and biochemical characteristics (morphology, urease test, oxidase test) before harvesting.

Preparation of the tested solution: All necessary quantities of compounds were weighed separately using a precision balance under sterile conditions and then dissolved in sterile distilled water. The tubes are then wrapped in aluminum foil to avoid exposure to light.

Gavage: Following *H. pylori* (PremSS1) infection, a variety of compounds including ADEF, amoxicillin, and pantoprazole were orally administered to mice for 14 consecutive days. These compounds were administered individually or in combination with a single dose. Amoxicillin was chosen for this study due to its frequent use in treating *H. pylori* as part of the triple therapy regimen, while pantoprazole, known for its low toxicity, was commonly employed in hospital settings.

Practically, the dose of compounds was prepared as follows: (i) pantoprazole (PPI) 150 mg/kg; (ii) amoxicillin (Amox) 30 mg/kg and (iii) ADEF 40 mg/kg.

Group-1  (n = 10): mice not infected (NI)
Group-2  (n = 10): mice infected with PremSS1
Group-3  (n = 10): mice infected with PremSS1 + PPI
Group-4  (n = 10): mice infected with PremSS1 + Amox
Group-5  (n = 10): mice infected with PremSS1 + PPI + Amox
Group-6  (n = 10): mice infected with PremSS1 + ADEF
Group-7  (n = 10): mice infected with PremSS1 + PPI + ADEF
Group-8  (n = 10): mice infected with PremSS1 + PPI + ADEF + Amox

At the conclusion of the experiment, mice from each group were euthanized in order to assess the antibacterial impact.

Euthanasia: Mice were euthanized by cervical dislocation and underwent laparotomy the day following the final day of treatment. The stomach was carefully isolated and excised by making a close incision near the esophagus and duodenum. Using a small curved-end scissor, the stomach was opened along the large curvature and placed in a Petri dish containing a small amount of physiological saline to remove any residual food. Subsequently, the stomach was bisected along the large curvature, from the duodenum to the esophagus, and then divided again along the small curvature. The right half of the stomach—devoid of the cardia—was further divided, with the first half being introduced into a tube containing physiological saline for bacteriological culture and molecular analysis, while the second half was placed in a dry tube and stored at −80 °C for subsequent complementary experiments. All procedures were conducted in strict adherence to the relevant animal ethics regulations.

- Bacterial culture to enumerate viable count of *H. pylori*

Each quarter of the mouse stomach was placed in a tube that was RNASE and DNASE free, containing 200 μL of physiological water. The tube, with the stomach piece, was then weighed to determine its mass before being ground using a sterile pestle. Next, a 10 µL portion was evenly spread onto an entire box of GSSA agar, which is Wilkins–Chalgren medium enriched with 10% human blood and made selective by adding vancomycin, trimethoprim, amphotericin B, bacitracin, polymyxin B, nalidixic acid, and cefsulodin. To facilitate spreading, 100 μL of physiological water was first deposited in the center of the box. Thereafter, subsequent dilutions (10^1^–10^3^) of 100 µL were prepared in GSSA medium. The remaining 90 µL were extracted for *H. pylori* quantification using qPCR. Control samples consisted of pure crushed stomachs obtained from uninfected mice. The Petri dishes containing the diluted samples were then incubated at 35 °C under microaerobic conditions. *H. pylori* colonies were identified based on their phenotypic and biochemical characteristics, including morphology, urease test, and oxidase test. After an incubation period of at least 5 days, two independent experimenters performed colony counts on the Petri dishes. All samples were prepared in duplicate, and the results were expressed as cfu/mg of stomach tissue.

- Relative quantification of *H. pylori* by qPCR

Automated extraction: After seeding part of the crushed stomachs, the remaining was used to extract the total DNA with the MagNA Pure 96 extractor system (Roche Diagnostics™) using the MagNA Pure 96 DNA kit and Viral NA Small Volume Kit following the manufacturer’s recommendations. For each ground material, the extracted DNA was recovered in 100 μL of elution buffer.

Quantitative PCR: The presence of *H. pylori* DNA and mouse housekeeping genes was quantified in the extracts by real time qPCR in detecting the fluorescence emitted by the neo-PCR products formed using SYBR Green™ (ABclonal, Woburn, MA, USA).

The specific amplification of *H. pylori* was carried out using a pair of primers targeting the gene coding for 23S rRNA, present in two copies in *H. pylori*. A 267-bp fragment of the 23S rRNA gene of *H. pylori* was amplified by using primers HPY-S and HPY-A. The primers were analyzed for 3′-terminal specificity to assure that they were specific to *H. pylori*. The sequences of these primers were:

- HPY-S (AGGTTAAGAGGATGCGTCAGTC) (SEQ ID NO:5);
- HPY-A (CGCATGATATTCCCATTAGCAGT) (SEQ ID NO:6).

These sequences correspond to nucleotides 1931 to 1952 and 2197 to 2175, respectively, of the 23S rRNA gene of *H. pylori* (GenBank accession number U27270).

The quantification of *H. pylori* is normalized via the mouse reference genes Gapdh and beta-actin. The primers used were:

- mGapdh1for (CTGCAGGTTCTCCACACCTATG) (SEQ ID NO:1);
- mGapdh1rev (GAATTTGCCGTGAGTGGAGTC) (SEQ ID NO:2);
- mActb2for (GACAGGATGCAGAAGGAGATTACTG) (SEQ ID NO:3);
- mActb2rev (ACATCTGCTGGAAGGTGGACA) (SEQ ID NO:4).

A volume of 5 μL of DNA was added to 20 μL of reaction mixture (primers 0.4 μM, Master Mix 2X supplied by the “LightCycler^®^ 480 SYBR Green I Master” kit from Roche Diagnostics^®^) at 20 ng/μL to amplify the gene coding for 23S rRNA and at 2 ng/µL to amplify the reference genes. The amplification was carried out in the LC480^®^ (Roche Diagnostics^®^).

The ratio of standard curves obtained from the DNA number of murine cells and that of bacteria were performed to quantify the number of murine cells (murine line MSCR) and bacteria in the sample. All graphs were represented in box plots prepared using GraphPad Prism-7 software (GraphPad Software Inc., La Jolla, CA, USA). Significant differences between untreated infected mice and different experimental conditions were compared using the Mann–Whitney test.

## 4. Conclusions

ADEF offers an alternative to antibiotics like clarithromycin, metronidazole, and levofloxacin, which are currently facing strong resistance from *H. pylori*. The proposed combination formulation includes ADEF, Amox, and PPI administered orally once a day for 14 days, proving to be an effective and safe strategy. This study emphasizes the importance of artesunate’s solubility in gastric acid, which plays a crucial role in achieving successful treatment, while highlighting the indispensability of PPI in formulation therapy.

Moreover, ADEF is an affordable and easily manufacturable option. Since artesunate is already approved by WHO and FDA for malaria treatment, developing ADEF as a promising candidate against *H. pylori* infections could potentially follow a faster development route. However, these preclinical findings must be verified in a clinical setting to determine the molecule’s efficacy as a valuable anti-*H. pylori* agent and a gastric anti-ulcer principle.

## Figures and Tables

**Table 1:** Plasmatic artesunate determination after oral administration of native artesunate and artesunate dry emulsion formulation (ADEF) in Sprague–Dawley rats.

Tested Item	Designation	Dose (mg/kg)	Cmax (ng/mL)	Tmax (min)	t½ (min)	AUC0-Tlast (h.ng/mL)
ADEF	Low dose-1	25	81	5	3.4	24.4
ADEF	Low dose-2	50	390	5	6.3	91.3
ADEF	Mid Dose	100	759	5	15.0	140.0
ADEF	High Dose	150	1890	5	20.0	402.0
Native Artesunate	High Dose	150	701	15	18	211.0

Cmax, maximum artesunate concentration; Tmax, time to reach Cmax; t½, terminal elimination half-life; AUC0-tlast, area under the concentration/time curve from the time of dosing to the last quantifiable concentration.

**Table 2:** Plasmatic dihydroartemisinin (DHA) determination after oral administration of native artesunate and artesunate dry emulsion formulation (ADEF) in Sprague Dawley rats**.**

Tested Item	Designation	Dose (mg/kg)	Cmax (ng/mL)	Tmax (min)	t½ (min)	AUC0-Tlast (h.ng/mL)
ADEF	Low dose-1	25	175	10	10.3	53.2
ADEF	Low dose-2	50	689	10	10.3	338.0
ADEF	Mid Dose	100	1320	15	17.6	546.0
ADEF	High Dose	150	3870	15	27.5	1790.0
Native Artesunate	High Dose	150	1450	45	22.0	728.0

Cmax, maximum dihydroartemisinin concentration; Tmax, time to reach Cmax; t½, terminal elimination half-life; AUC0-tlast, area under the concentration/time curve from the time of dosing to the last quantifiable concentration.

## Data Availability

Not applicable.

## Supplementary Materials

The supporting information can be downloaded at: https://www.mdpi.com/article/10.3390/ijms241311008/s1.

## Abbreviations

ADEF: artesunate dry emulsion formulation; Amox, amoxicillin; PPI, proton pump inhibitor; DHA, dihydroartemisinin; SGF, simulated gastric fluid; CMC, carboxymethylcellulose; PremSS1, pre-murine Sydney strain-1; Cmax, maximum plasma concentration; Tmax, time to reach Cmax; t½, terminal elimination half-life; AUC0-tlast, area under the concentration/time curve from the time of dosing to the last quantifiable concentration.

## References

[1] Hooi J.K., Lai W.Y., Ng W.K., Suen M.M., Underwood F.E., Tanyingoh D., Malfertheiner P., Graham D.Y., Wong V.W., Wu J.C. (2017). Global prevalence of Helicobacter pylori infection: Systematic review and meta-analysis. Gastroenterology.

[2] Tempera P.J., Michael M., Tageldin O., Hasak S. (2022). Gastric Cancer Due to Chronic H. pylori Infection: What We Know and Where We Are Going. Diseases.

[3] Mubaraki M.A., Alalhareth A.S., Aldawood E., Albouloshi A., Aljarah M.S., Hafiz T.A., Alkhudhayri A., Thagfan F.A., El-khadragy M.F., Al-Megrin W.A. (2022). The iron deficiency anemia in association to Helicobacter pylori infection in Najran city, Saudi Arabia. J. King Saud Univ.-Sci..

[4] Koseki M., Sheu M.J., Tsai K.-T., Ho C.-H., Liu H.-H., Lin H.-J., Lin C.-L., Huang C.-C. (2023). Eradication therapy may decrease the risk of immune thrombocytopenia after Helicobacter pylori infection: A retrospective cohort study in Taiwan. BMC Gastroenterol..

[5] Suzuki S., Kusano C., Horii T., Ichijima R., Ikehara H. (2022). The ideal Helicobacter pylori treatment for the present and the future. Digestion.

[6] Kocsmár É., Buzás G.M., Szirtes I., Kocsmár I., Kramer Z., Szijártó A., Fadgyas-Freyler P., Szénás K., Rugge M., Fassan M. (2021). Primary and secondary clarithromycin resistance in Helicobacter pylori and mathematical modeling of the role of macrolides. Nat. Commun..

[7] Azadbakht S., Moayyedkazemi A., Azadbakht S., Fard S.A., Soroush S. (2022). Evaluation of antibiotic resistance of Helicobacter pylori bacteria obtained from gastric biopsy samples: A cohort study. Ann. Med. Surg..

[8] Caliskan R., Tokman H.B., Erzin Y., Saribas S., Yuksel P., Bolek B.K., Sevuk E.O., Demirci M., Yılmazli O., Akgul O. (2015). Antimicrobial resistance of Helicobacter pylori strains to five antibiotics, including levofloxacin, in Northwestern Turkey. Rev. Da Soc. Bras. De Med. Trop..

[9] Ho J.J.C., Argueta E.A., Moss S.F. (2022). Helicobacter pylori Treatment Regimens: A US Perspective. Gastroenterol. Hepatol..

[10] Korona-Glowniak I., Glowniak-Lipa A., Ludwiczuk A., Baj T., Malm A. (2020). The in vitro activity of essential oils against Helicobacter pylori growth and urease activity. Molecules.

[11] Sisto F., Scaltrito M.M., Masia C., Bonomi A., Coccè V., Marano G., Haynes R.K., Miani A., Farronato G., Taramelli D. (2016). In vitro activity of artemisone and artemisinin derivatives against extracellular and intracellular Helicobacter pylori. Int. J. Antimicrob. Agents.

[12] Su T., Li F., Guan J., Liu L., Huang P., Wang Y., Qi X., Liu Z., Lu L., Wang D. (2019). Artemisinin and its derivatives prevent Helicobacter pylori-induced gastric carcinogenesis via inhibition of NF-κB signaling. Phytomedicine.

[13] Cheong D.H., Tan D.W., Wong F.W., Tran T. (2020). Anti-malarial drug, artemisinin and its derivatives for the treatment of respiratory diseases. Pharmacol. Res..

[14] World Health Organization (2006). Guidelines for the Treatment of Malaria.

[15] Chadha R., Gupta S., Pathak N. (2012). Artesunate-loaded chitosan/lecithin nanoparticles: Preparation, characterization, and in vivo studies. Drug Dev. Ind. Pharm..

[16] Drugbank Artesunate. http://www.iupac.org/dhtml_home.html.

[17] Haynes R.K., Chan H.W., Lung C.M., Ng N.C., Wong H.N., Shek L.Y., Williams I.D., Cartwright A., Gomes M.F. (2007). Artesunate and dihydroartemisinin (DHA): Unusual decomposition products formed under mild conditions and comments on the fitness of DHA as an antimalarial drug. ChemMedChem Chem. Enabling Drug Discov..

[18] Chinaeke E., Chime S., Onyishi V., Attama A., Okore V. (2015). Formulation development and evaluation of the anti-malaria properties of sustained release artesunate-loaded solid lipid microparticles based on phytolipids. Drug Deliv..

[19] Prashanth G.P., Maralihalli M.B., Bagalkot P.S., Joshi S.N. (2012). Intravenous artesunate for transfusion-transmitted Plasmodium vivax malaria in a preterm neonate. Pediatrics.

[20] Parapini S., Olliaro P., Navaratnam V., Taramelli D., Basilico N. (2015). Stability of the antimalarial drug dihydroartemisinin under physiologically relevant conditions: Implications for clinical treatment and pharmacokinetic and in vitro assays. Antimicrob. Agents Chemother..

[21] Chung I.-Y., Jang H.-J., Yoo Y.-J., Hur J., Oh H.-Y., Kim S.-H., Cho Y.-H. (2022). Artemisinin displays bactericidal activity via copper-mediated DNA damage. Virulence.

[22] Goswami S., Bhakuni R.S., Chinniah A., Pal A., Kar S.K., Das P.K. (2012). Anti-Helicobacter pylori potential of artemisinin and its derivatives. Antimicrob. Agents Chemother..

[23] Tanner J.-A., Tyndale R.F. (2017). Variation in CYP2A6 activity and personalized medicine. J. Pers. Med..

[24] Scott D.R., Sachs G., Marcus E.A. (2016). The role of acid inhibition in Helicobacter pylori eradication. F1000Research.

[25] FDA. Food and Drug Administration FDA Approves Only Drug in U.S. to Treat Severe Malaria. https://www.fda.gov/news-events/press-announcements/fda-approves-only-drug-us-treat-severe-malaria.

[26] Tao A., Song Z., Feng X., Zhang A., He H., Chen Y. Antibacterial and antiviral activities of Artemisia Annua aqueous extract in vitro. Proceedings of the IOP Conference Series: Earth and Environmental Science.

[27] Myran L., Zarbock S.D. (2018). Management of Helicobacter pylori infection. US Pharm..

[28] Roberts L.T., Issa P.P., Sinnathamby E.S., Granier M., Mayeux H., Eubanks T.N., Malone K., Ahmadzadeh S., Cornett E.M., Shekoohi S. (2022). Helicobacter Pylori: A Review of Current Treatment Options in Clinical Practice. Life.

[29] Mohsen S., Dickinson J.A., Somayaji R. (2020). Update on the adverse effects of antimicrobial therapies in community practice. Can. Fam. Physician.

[30] Gisbert J.P. (2020). Rifabutin for the treatment of Helicobacter pylori infection: A review. Pathogens.

[31] Savoldi A., Carrara E., Graham D.Y., Conti M., Tacconelli E. (2018). Prevalence of antibiotic resistance in Helicobacter pylori: A systematic review and meta-analysis in World Health Organization regions. Gastroenterology.

[32] Borraccino A.V., Celiberto F., Pricci M., Girardi B., Iannone A., Rendina M., Ierardi E., Di Leo A., Losurdo G. (2022). Rifabutin as salvage therapy for Helicobacter pylori eradication: Cornerstones and novelties. World J. Gastroenterol..

[33] Elvers K.T., Wilson V.J., Hammond A., Duncan L., Huntley A.L., Hay A.D., Van Der Werf E.T. (2020). Antibiotic-induced changes in the human gut microbiota for the most commonly prescribed antibiotics in primary care in the UK: A systematic review. BMJ Open.

[34] Patangia D.V., Anthony Ryan C., Dempsey E., Paul Ross R., Stanton C. (2022). Impact of antibiotics on the human microbiome and consequences for host health. MicrobiologyOpen.

[35] Zhou X., Sun W.-J., Wang W.-M., Chen K., Zheng J.-H., Lu M.-D., Li P.-H., Zheng Z.-Q. (2013). Artesunate inhibits the growth of gastric cancer cells through the mechanism of promoting oncosis both in vitro and in vivo. Anti-Cancer Drugs.

[36] Denny J.E., Schmidt N.W. (2019). Oral Administration of Clinically Relevant Antimalarial Drugs Does Not Modify the Murine Gut Microbiota. Sci. Rep..

