# Supplementary material for: Artesunate Dry Emulsion Formulation Combined with Antibiotics for Treatment of Helicobacter pylori Infections: In Vitro/In Vivo Evaluation
Source: Int J Mol Sci. 2023 Jul 2;24(13):11008. doi: 10.3390/ijms241311008 (PMC10342150; doi:10.3390/ijms241311008)
Supplement: Supplementary file 1 [file ijms-24-11008-s001.zip › ijms-2408367-supplementary.pdf]

Table-S1. Liquid chromatographic mass spectrometric (LC/MS/MS) method summary for the quantification of Artesunate (ART) and Dihydroartemisinin (DHA) in rat plasma.

**Parameters**	**Method**
Matrix for calibration standards and quality control samples:	Rat K_3-EDTA plasma
Calibration range:	1.00 – 500.00 ng/mL of ART<br>1.00 – 500.00 ng/mL of DHA
Lower limit of quantification (LLOQ):	1.00 ng/mL
Upper limit of quantification (ULOQ):	500.00 ng/mL
Analytical column:	Poroshell 120, EC-C18, 50 x 4.6 mm, 2.7 µm (Agilent)
Mobile phase A:	2 mM ammonium acetate with 0.02% acetic acid
Mobile phase B:	Methanol
Gradient elution	68.0 – 95.0% mobile phase B
Flow rate:	1.00 mL/min
Injection volume:	12 µL
Analysis time:	4.00 minutes
Ionisation mode:	Positive ion mode
Sample volume:	25 µL
Sample preparation:	Protein precipitation (25 fold dilution)
LC-MS/MS system:	Shimadzu 20AC and API 5000
Analytes:	ART and DHA
Internal standard:	ART-d_4

Table-S2. Precision and accuracy results for Artesunate quantification

Intra-Assay	Analytical Run No.	Quality Control Samples ng/mL			Calibration Standards ng/mL							
		LLOQ	QC A	QC C	Std B	Std C	Std D	Std E	Std F	Std G	Std H	Std I
		1.00	3.00	400.00	1.00	2.00	10.00	50.00	150.00	250.00	350.00	500.00
1	PV03	1.05	2.66	400.49	0.96	1.92	10.09	51.83	150.09	254.92	329.36	503.12
		0.84	2.51	391.57	1.05	1.98	10.95	50.95	151.84	249.39	309.72	506.96
		1.03	2.82	378.77								
		0.97	2.79	377.38								
	%C.V	9.7	5.3	2.8								
	% Bias	-2.8	-10.2	-3.4								
2	PV04	1.06	2.89	373.11	1.02	1.96	10.44	51.30	151.76	244.68	339.62	484.65
		0.97	2.92	382.05	0.92	2.22	10.53	50.30	149.30	253.92	334.80	469.47
		1.03	2.91	362.94								
		1.16	2.83	379.40								
	%C.V	7.5	1.4	2.3								
	% Bias	5.5	-3.7	-6.4								
Inter-Assay	**Mean**	**1.014**	**2.791**	**380.714**	**0.988**	**2.020**	**10.503**	**51.095**	**150.748**	**250.728**	**328.375**	**491.050**
	**S.D**	**0.0921**	**0.1410**	**11.3458**	**0.0585**	**0.1356**	**0.3536**	**0.6417**	**1.2578**	**4.6950**	**13.1239**	**17.3731**
	**%C.V**	**9.1**	**5.1**	**3.0**	**5.9**	**6.7**	**3.4**	**1.3**	**0.8**	**1.9**	**4.0**	**3.5**
	**n**	**8**	**8**	**8**	**4**	**4**	**4**	**4**	**4**	**4**	**4**	**4**
	**% Bias**	**1.4**	**-7.0**	**-4.8**	**-1.3**	**1.0**	**5.0**	**2.2**	**0.5**	**0.3**	**-6.2**	**-1.8**
	%C.V = Percent Coefficient of Variation<br>S.D = Standard Deviation											

Curve Parameter Summary **Weighted Least Squares Regression Equation: y = ax + b**

Analytical Run No.	r	a	b
PV03	0.9985	0.094	0.00575
PV04	0.9986	0.0915	0.0014
**Mean**	0.99855	0.09275	0.003575

Table-S3. Precision and accuracy results for Dihydroartemisinin quantification

Intra-Assay	Analytical Run No.	Quality Control Samples ng/mL			Calibration Standards ng/mL							
		LLOQ	QC A	QC C	Std B	Std C	Std D	Std E	Std F	Std G	Std H	Std I
		1.00	3.00	400.00	1.00	2.00	10.00	50.00	150.00	250.00	350.00	500.00
1	PV03	1.05	2.73	407.47	0.94	1.87	9.77	49.85	153.66	254.61	326.69	503.99
		0.99	2.61	396.18	1.06	2.07	11.00	50.68	151.58	255.30	314.24	510.52
		1.07	3.00	381.92								
		0.97	2.95	382.38								
	%C.V	4.7	6.5	3.1								
	% Bias	2.0	-5.9	-2.1								
2	PV04	1.13	3.05	386.37	1.00	2.24	10.82	52.50	156.96	247.67	345.95	488.64
		1.18	3.13	374.50	0.97	1.86	10.10	49.66	151.32	248.15	326.72	455.93
		0.96	3.07	362.41								
		1.01	2.91	375.47								
	%C.V	9.6	3.1	2.6								
	% Bias	7.0	1.3	-6.3								
Inter-Assay	**Mean**	**1.045**	**2.931**	**383.338**	**0.993**	**2.010**	**10.423**	**50.673**	**153.380**	**251.433**	**328.400**	**489.770**
	**S.D**	**0.0786**	**0.1781**	**13.8223**	**0.0512**	**0.1813**	**0.5835**	**1.2963**	**2.6063**	**4.0819**	**13.0927**	**24.3529**
	**%C.V**	**7.5**	**6.1**	**3.6**	**5.2**	**9.0**	**5.6**	**2.6**	**1.7**	**1.6**	**4.0**	**5.0**
	**n**	**8**	**8**	**8**	**4**	**4**	**4**	**4**	**4**	**4**	**4**	**4**
	**% Bias**	**4.5**	**-2.3**	**-4.2**	**-0.8**	**0.5**	**4.2**	**1.3**	**2.3**	**0.6**	**-6.2**	**-2.0**

%C.V = Percent Coefficient of Variation
S.D = Standard Deviation

Curve Parameter Summary — **Weighted Least Squares Regression Equation: y = ax + b**

Analytical Run No.	r	a	b
PV03	0.9983	0.0346	0.00419
PV04	0.9981	0.0378	0.00235
**Mean**	0.99820	0.03620	0.003270
